# Recent Avian Influenza Virus A/H5N1 Evolution in Vaccinated and Unvaccinated Poultry from Farms in Southern Vietnam, January–March 2010

**DOI:** 10.1111/j.1865-1682.2011.01229.x

**Published:** 2011-12

**Authors:** N T Long, T T Thanh, H R van Doorn, P P Vu, P T Dung, T T K Dung, T N Tien, D T T Thao, P Hung, N V Quang, N T Hoa, J E Bryant, M F Boni

**Affiliations:** 1 Regional Animal Health Office No. 6Ho Chi Minh City, Vietnam; 2 Oxford University Clinical Research UnitHo Chi Minh City, Vietnam; 3 Nuffield Department of Medicine, Centre for Tropical Medicine, University of OxfordOxford, UK; 4 Southeast Asian Infectious Diseases Clinical Research NetworkJakarta, Indonesia; 5 Regional Animal Health Office No. 7Can Tho, Vietnam; 6 Central Viet Nam Veterinary InstituteNha Trang, Vietnam; 7 MRC Centre for Genomics and Global Health, University of OxfordOxford, UK

**Keywords:** emerging diseases, vaccine, veterinary epidemiology, zoonosis/zoonotics, virus

## Abstract

We report 15 new avian influenza virus A/H5N1 haemagglutinin (HA) sequences sampled from visibly sick domestic poultry in southern Vietnam, between 1 January 2010 and 6 March 2010. These HA sequences form a new sub-clade of the clade 1 H5N1 viruses that have been circulating in Vietnam since 2003/2004. The viruses are characterized by a change from isoleucine to valine at position 514 (I514V) and are 1.8% divergent at the nucleotide level from HA sequences sampled in Vietnam in 2007. Five new amino acid changes were observed at previously identified antigenic sites, and three were located within structural elements of the receptor-binding domain. One new mutation removed a potential N-linked glycosylation site, and a methionine insertion was observed in one virus at the polybasic cleavage site. Five of these viruses were sampled from farms where poultry were vaccinated against H5N1, but there was no association between observed amino acid changes and flock vaccination status. Despite the current lack of evidence for antigenic drift or immune escape in Vietnamese H5N1 viruses, continued surveillance remains a high priority.

Over 500 human cases of H5N1 influenza virus infection have been recorded since the emergence of this highly pathogenic virus subtype in 1996/1997 (Peiris et al., 2007; Tarantola et al., 2010). Although the global incidence of human H5N1 cases has declined recently, surveillance and molecular characterization of highly pathogenic avian influenza (HPAI) viruses remain a high priority. The majority of human cases have occurred in three countries, Indonesia (174), Egypt (130) and Vietnam (119); all of which have high rates of household poultry ownership, a large network of markets with high levels of poultry sales and consumption (Domenech et al., 2009) and frequent outbreaks and high prevalence of HPAI in domestic poultry. Vietnam is home to more than 220 million domestic poultry, with more than 40% of households owning chickens or ducks. H5N1 avian influenza has been a constant threat as a potentially fatal zoonotic infection, causing regular outbreaks between December and February every year. Although the majority of human H5N1 cases in Vietnam occurred in 2004 and 2005, the past 2 years have seen 12 cases including seven fatalities.

The global phylogenetic structure of avian H5N1 viruses is described by WHO-defined clades and sub-clades based on haemagglutinin (HA) sequences (WHO/OIE/FAO H5N1 Evolution Working Group, 2009). Vietnamese H5N1 viruses comprise clade 1, clade 2.3.2 and clade 2.3.4 (Le et al., 2008; Nguyen et al., 2008). Clade 7 viruses were introduced across the Vietnamese–Chinese border in 2008 (Nguyen et al., 2009), but have not yet become established in Vietnam. To date, H5N1 circulation in southern Vietnam has been primarily, but not exclusively, limited to clade 1 viruses (Wan et al., 2008). The genetic structure of H5N1 virus populations in Vietnam suggests that gene flow is limited between northern and southern Vietnam (Smith et al., 2006a).

Since October 2005, the Vietnamese government has pursued ambitious biannual mass vaccination campaigns for domestic poultry (Hinrichs et al., 2010). This vaccination strategy is one of the important explanatory variables of the reduced frequency of outbreaks and lower overall levels of infection (Domenech et al., 2009; Henning et al., 2009). Approximately 250–350 million poultry are vaccinated in March/April and August/September each year with an inactivated vaccine generated by reverse genetics; the current formulation (Re-1) contains the HA and neuraminidase of A/Goose/Guangdong/1/96. Re-1 is now only distantly related to currently circulating viruses; however, it provides good protection against contemporary clade 1, 2.2 and 2.3.4 viruses (Tian et al., 2010). In 2011, Vietnam plans to update the vaccine strain to Re-5 (A/duck/Anhui/1/2006), a clade 2.3.4 virus more closely related to Chinese circulating strains (Chen, 2009). Monitoring genetic diversity and ongoing evolution in vaccinated populations is critical for identifying changes in virulence and immune escape, but surveillance is compromised by the fact that some chickens seroconvert without showing signs of disease (Henning et al., 2010). One explanatory hypothesis for the lack of clinical signs posits the circulation of H5N1 variants exhibiting low pathogenicity in chickens; however, virological evidence to confirm this is not yet available.

## Methods

We sequenced the HA of H5N1 viruses obtained from diseased domestic poultry during passive surveillance activities of January–March 2010 in four provinces of southern Vietnam: Khanh Hoa, Ben Tre, Soc Trang and Ca Mau.

Sampling was conducted by veterinary technicians from the respective provincial sub-departments of animal health as per national guidelines in response to suspected HPAI outbreaks. Cloacal specimens were collected from one to three birds per flock and screened by RT-PCR using OIE standard protocols (OIE, 2008). Sixteen farms reported diseased poultry during the 3-month study period, and 17 samples collected from these farms tested H5-positive. Four farms reported a history of vaccination within the previous 3 months. Direct sequencing of HA was attempted on all samples as previously described (Guan et al., 2002), and 15 HA sequences were successfully generated (9 from meat ducks, 4 from chickens and one each from a quail and Muscovy duck; Genbank accession numbers CY081026–CY081040).

**Fig. 1:**
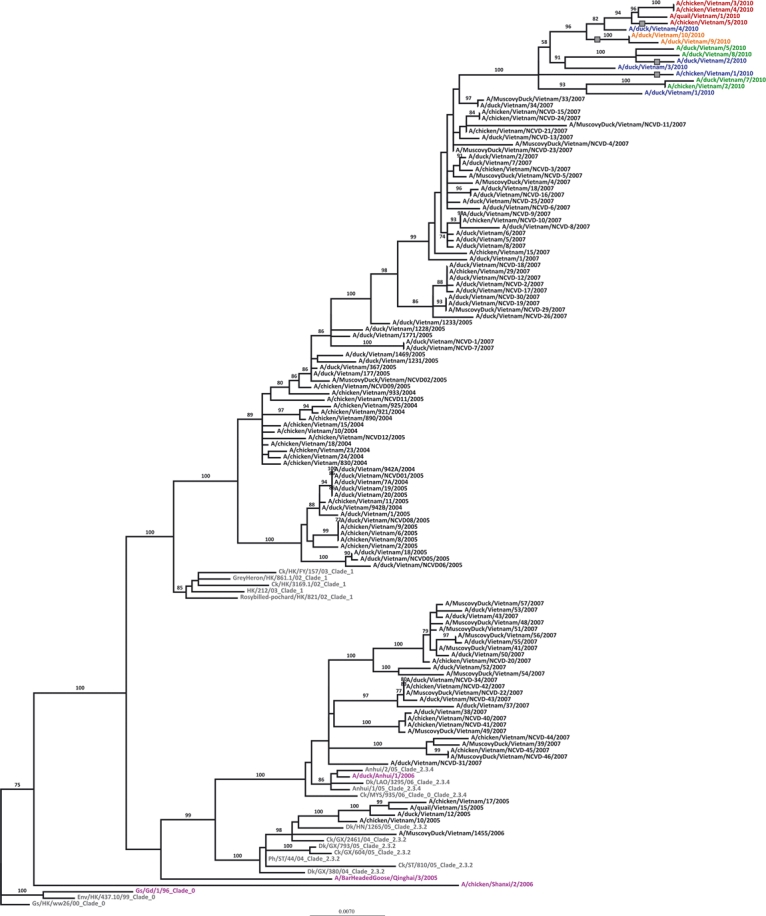
Maximum-likelihood phylogenetic tree of 142 Vietnamese avian H5N1 HA sequences. Tree rooted on Gs/Gd/1/96. Only sequences available through the Influenza Genome Sequencing Project (National Institutes of Health, USA) were included in the analysis. Bootstrap values ≥70% are shown on the branches. Sequences from 2010 are colour-coded by location: Khanh Hoa (red), Ben Tre (orange), Soc Trang (green) and Ca Mau (blue). Grey boxes on branches denote the five sequences that were sampled from vaccinated farms. Vaccine strains used in China (Chen, 2009) shown in magenta. Model of evolution used by RAxML: GTR-Γ, α = 0.422679, AC = 1.149255, AG = 5.429345, AT = 0.628623, CG = 0.232736, CT = 7.840430, GT = 1.0.

To place the 15 new HA sequences in regional context, we downloaded all HA sequences (>1600 nt) of Vietnamese avian H5N1 viruses from GenBank (*n* = 105; 33 chicken, 71 duck and 1 quail), only including sequences from the Influenza Genome Sequencing Project (IGSP) (Ghedin et al., 2005) as the quality control and sequencing redundancy in the IGSP makes it more likely that these sequences are free of sample contamination and sequencing errors (Boni et al., 2010). Our 15 HA sequences were aligned with the 105 GenBank sequences, 16 clade markers, three clade 0 sequences as outgroups and three vaccine strains used in China (Chen, 2009), which are the most likely vaccine candidates to be considered for use in Vietnam. Alignments were performed with MUSCLE v3.8 (Edgar, 2004), and maximum-likelihood phylogenetic inference was performed with RAxML using 1000 bootstrap replicates (Stamatakis, 2006; Stamatakis et al., 2008). Figure 1 shows the phylogeny of all 142 sequences.

**Fig. 2:**
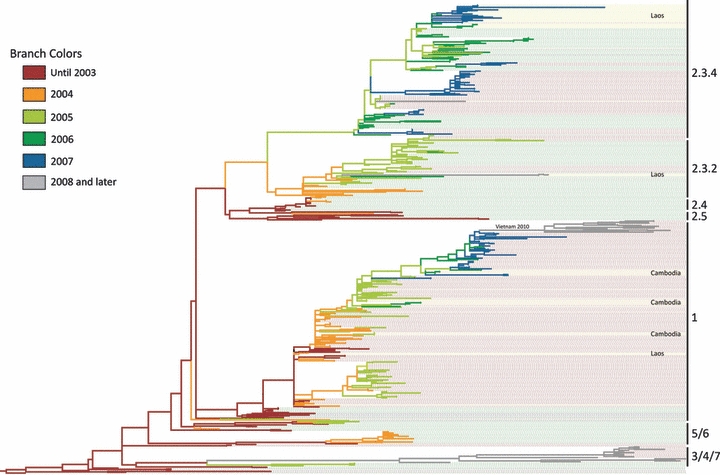
Maximum-likelihood phylogenetic tree of 574 H5N1 HA sequences from SE Asia: Vietnam, Laos, Cambodia (all years) and Yunnan, Guangxi and Guangdong provinces in China (2003 and later). Tree rooted on Gs/Gd/1/96. Branch colours denote year of sampling. The shaded areas to the right of the branch tips correspond to the country where the virus was sampled: Vietnam (red), China (green), Laos (yellow) and Cambodia (orange). Numbers at the right hand edge of the figure denote WHO-defined clades. Model of evolution used by RAxML: GTR-Γ, α = 0. 477645, AC = 0.878452, AG = 5.045729, AT = 0.606001, CG = 0.176209, CT = 7.067432, GT = 1.0.

To investigate whether sequences outside Vietnam may be related to the 2010 strains sequenced in this study, we used a broader set of sequences to infer a second phylogenetic tree using both IGSP and non-IGSP sequences. We downloaded all subtype H5N1 HA sequences (>1600 nt) from Cambodia (*n* = 25), Laos (*n* = 36), Vietnam (*n* = 293), the two Chinese provinces of Yunnan (*n* = 68) and Guangxi (*n* = 78) that border Vietnam, and the Chinese province of Guangdong (*n* = 37) that is close to Vietnam and an important epicentre of avian H5N1 circulation. Chinese sequences sampled before 2003 were excluded to reduce the size of the data set. These sequences were aligned with clade markers, vaccine strains and outgroups as above. Figure 2 shows a maximum-likelihood phylogenetic tree (RAxML, 100 bootstrap replicates) of these 574 sequences.

## Results and Discussion

The fifteen 2010 sequences formed a clear bootstrap-supported sub-clade within the clade 1 sequences, with a mean distance of 31 nt (1.8%) from the ancestral 2007 viruses (Figure 1). The 2010 clade is characterized by a substitution from isoleucine to valine at position 514 (I514V; H5 numbering as in WHO Global Influenza Program (2005) and Smith et al. (2006a)), with four new amino acid changes observed in at least two sequences (Table 1). Two previously polymorphic sites (K48R and N168D) exhibited dramatic increases in frequency in the 2010 clade as compared to the 2007 clade. Five new amino acid changes were located in previously identified antigenic sites (T36A, D45G, P136S, S155N/G and A185E) (WHO Global Influenza Program, 2005; Stevens et al., 2006). Three amino acid changes (A184G, A185E and A214S) occurred within predicted loop structures of the receptor-binding domain (Stevens et al., 2006), and the S155N/G mutations remove a potential N-linked glycosylation site (Smith et al., 2006a; Yen et al., 2009). One virus had a single methionine insertion at the polybasic cleavage site. The phenotypic effects of these changes will be characterized in future analyses.

**Table 1:** 15 HA sequences sampled during January–March 2010 in southern Vietnam

Sequence name	Date of collection	Location (province)	New amino acid changes from clade 1 2007 sequences
A/Chicken/Vietnam/1/2010*	Jan 1	Ca Mau	**I514V**, A214S
A/Duck/Vietnam/1/2010	Jan 7	Ca Mau	**I514V**, **A185E**, **N309T**, D45G, R458K
A/Duck/Vietnam/2/2010*	Jan 10	Ca Mau	**I514V**
A/Duck/Vietnam/3/2010	Jan 17	Ca Mau	**I514V**, S155G, N236S; insertion (-)326M
A/Duck/Vietnam/4/2010†	Jan 24	Ca Mau	T204M
A/Duck/Vietnam/5/2010†	Jan 25	Soc Trang	S155N
A/Chicken/Vietnam/2/2010	Feb 2	Soc Trang	**I514V, N309T**
A/Duck/Vietnam/8/2010	Feb 12	Soc Trang	**I514V**, **A185E**, V200I
A/Duck/Vietnam/7/2010	Feb 17	Soc Trang	**I514V**, **N309T**, A184G
A/Chicken/Vietnam/4/2010	Feb 23	Khanh Hoa	**I514V**
A/Quail/Vietnam/1/2010	Feb 23	Khanh Hoa	**I514V**, C42W, K161R
A/Chicken/Vietnam/3/2010	Feb 25	Khanh Hoa	**I514V**
A/Chicken/Vietnam/5/2010*	Feb 25	Khanh Hoa	**I514V**, I71T
A/Duck/Vietnam/9/2010*‡	Mar 6	Ben Tre	**I514V**, **T36A**, **P136S**, L41H, C42R
A/Duck/Vietnam/10/2010*‡	Mar 6	Ben Tre	**I514V**, **T36A**, **P136S**

* vaccinated farm.

† Not sequenced through position 514.

‡ Samples from the same farm.

Boldface amino acid changes observed in more than one virus.

When considered in a broader phylogenetic context – including IGSP and non-IGSP sequences from Vietnam, Cambodia, Laos and southern China – the 2010 Vietnamese HA sequences still form a bootstrap-supported (99%) sub-clade of the clade 1 viruses. Although it is likely that clade 1 viruses circulating in 2008 and 2009 were genetically close to the 2010 viruses described here, no Vietnamese H5N1 sequences from 2008 or 2009 are available in GenBank.

The 2010 viruses sampled in this report originated from chicken and duck farms with flock sizes ranging from 4 to >8000 birds. The outbreak farms experienced significant losses, with most farms experiencing between 10% and 45% flock mortality. Four of the 15 farms reported flock vaccination within the previous 3 months; however, we do not have access to more precise information or flock histories to document what percentage of the flock was vaccinated nor to confirm whether all ducks received the recommended two doses. It should be noted that even if sera had been collected from the outbreak farms, it would not have been possible to confirm vaccination status, because the current vaccine formulation in Vietnam (inactivated Re-1 unmarked virus) generates an immune response that is indistinguishable from natural infection with field virus. We strongly recommend that future outbreak response activities more precisely document the details of flock vaccination.

Many factors on both an individual and flock level may affect vaccine efficacy, such as problems with cold chain or poor vaccine quality, improper dosing (inappropriate age, failure to administer booster) or population turnover within the flock. Previous studies indicate that vaccination under field conditions may fail to achieve the same magnitude of immunity as observed in experimental validations (Henning et al., 2010). In particular, seroconversion is known to be particularly poor in ducks and requires a minimum of two doses, recommended at 7 and 21 days, for full protection (Henning et al., 2010). Thus, it is difficult to determine the true immune status and susceptibility to infection or outbreaks of any particular flock.

Our results concur with other reports suggesting that current vaccination campaigns are failing to provide adequate protection from infection and disease in vaccinated flocks and may thus be generating partially immune populations that could select for new antigenic variants of H5N1 (Smith et al., 2006b; Domenech et al., 2009; Henning et al., 2010). However, there is as yet no evidence that vaccination failure is the result of viral immune escape. Complete antigenic characterizations of the viruses sequenced in this study will be required to fully evaluate the phenotypic implications of the observed mutations. In addition, it must be emphasized that the presence of new amino acid changes described in the 2010 H5N1 Vietnamese viruses did not correlate with vaccination status; two of the viruses from vaccinated farms did not show new amino acid changes at antigenic sites.

The combination of aggressive vaccination campaigns and HPAI outbreak control measures in Vietnam has substantially reduced H5N1 virus circulation throughout Vietnam since the first outbreaks in 2003–2005. However, HPAI continues to circulate among poorly vaccinated flocks. As H5 avian viruses have demonstrated capacity for immune-escape evolution in the past (Lee et al., 2004; Domenech et al., 2009), it is clear that sustained and vigilant surveillance of field viruses remains critically important.
